# A survey of COVID-19 detection and prediction approaches using mobile devices, AI, and telemedicine

**DOI:** 10.3389/frai.2022.1034732

**Published:** 2022-12-02

**Authors:** John Shen, Siddharth Ghatti, Nate Ryan Levkov, Haiying Shen, Tanmoy Sen, Karen Rheuban, Kyle Enfield, Nikki Reyer Facteau, Gina Engel, Kim Dowdell

**Affiliations:** ^1^Department of Computer Science, University of Virginia, Charlottesville, VA, United States; ^2^School of Medicine, University of Virginia, Charlottesville, VA, United States; ^3^University of Virginia (UVA) Health System, University of Virginia, Charlottesville, VA, United States

**Keywords:** COVID-19 detection, mobile devices, AI, telemedicine, IoT

## Abstract

Since 2019, the COVID-19 pandemic has had an extremely high impact on all facets of the society and will potentially have an everlasting impact for years to come. In response to this, over the past years, there have been a significant number of research efforts on exploring approaches to combat COVID-19. In this paper, we present a survey of the current research efforts on using mobile Internet of Thing (IoT) devices, Artificial Intelligence (AI), and telemedicine for COVID-19 detection and prediction. We first present the background and then present current research in this field. Specifically, we present the research on COVID-19 monitoring and detection, contact tracing, machine learning based approaches, telemedicine, and security. We finally discuss the challenges and the future work that lay ahead in this field before concluding this paper.

## 1. Introduction

COVID-19, a virus from the coronavirus family, has taken center stage since it was first detected in 2019 (No, 2001). COVID-19 has impacted all aspects of our daily lives and has inherently changed what “normal” day to day life currently is. There have been a large amount of efforts over the past years on researching and engineering solutions to effectively combat the spread of COVID-19. One solution is to leverage mobile Internet of Thing (IoT) devices that already have been widely used in our daily lives.

Indeed, considering the popularity of mobile IoT devices used by the population and the advancement of sensing capabilities (e.g., respiratory rate) (as listed in Seshadri et al., 2020), mobile IoT devices become a promising approach for COVID-19 monitoring, detection and prediction. Wearable devices' sensor data can be used to alert people of a potential COVID-19 infection before symptoms become severe even for individuals who are asymptomatic (Seshadri et al., 2020).

Mobile IoT devices also enable people contact tracing in order to predict and control the spread of COVID-19, and alert people who have contacted infected people. Artificial Intelligence (AI) and machine learning (ML) techniques also have been used for COVID-19 detection. These techniques are used in mobile IoT devices or in the cloud to assist IoT devices to detect COVID-19 infection. In addition, as telemedicine can better protect patients and doctors from being infected and also reduce the diagnosis time of the doctors, the combination use of mobile devices and telemedicine is proposed to facilitate patients for visiting doctors remotely. Further, while an individual's data is collected for COVID-19 detection, his/her privacy should be protected. Thus, security is also an important aspect in the COVID-19 detection.

Given the significant amount of research efforts on using mobile IoT devices for COVID-19 detection and prediction, in this paper, we conduct a survey of a subset of the COVID-19 related work. Specifically, we categorize the related work into the following topics and accordingly present a survey of the current research efforts on using mobile IoT devices, AI and telemedicine for COVID-19 detection and prediction.

1. Detection and Monitoring,
2. Contact Tracing,
3. Machine Learning Framework and Approaches,
4. Telemedicine,
5. Security.

Table 1 shows the summary of the number of papers cited in each category in this paper.

**Table 1:** Summary of the number of papers cited in each category in this survey.

**Sections**	**Section 2.1**	**Section 2.2**	**Section 2.3**	**Section 2.4**	**Section 2.5**
Topic and # of references	Detection and monitoring (Awotunde et al., 2022)	Contact tracing (Andreas et al., 2021)	Machine learning framework and approaches (Gouissem et al., 2021)	Telemedicine (Almalki et al., 2022)	Security (Andreas et al., 2021)

The rest of the paper is organized as follows. Section 2 presents a survey of the current work being conducted on the aforementioned topics related to using mobile devices to detect or predict COVID-19. Section 3 discusses the challenges and future work that lay ahead in the field. Section 4 concludes the paper.

## 2. Survey of COVID-19 detection and prediction work

In this section, we present a survey of the current research being conducted on the topic of using mobile devices for COVID-19 detection and prediction. We classify the related work into five categories and present the work in each category in the following subsections. Table 2 shows an overview of all methods and papers reviewed in this survey according to their categories or sub-categories. As we cited more than 150 papers in this survey, we pick up 16 papers as an example to show the work in the area of COVID-19 detection and prediction using mobile devices, AI and telemedicine. A summary of the selected studies is presented in Table 3.

**Table 2:** An overview of all methods and papers reviewed in this survey.

**Section**	**Category**	**References**
Detection and monitoring		(Arun et al., 2020; Choyon et al., 2020; Dhadge and Tilekar, 2020; Ding et al., 2020; Hossain et al., 2020; Josephine et al., 2020; Karthi and Jayakumar, 2020; Seshadri et al., 2020; Swayamsiddha and Mohanty, 2020; Al-Emran and Ehrenfeld, 2021; Anjali et al., 2021; Ashwin et al., 2021; Awotunde et al., 2021, 2022; Chakkor et al., 2021a,b; Elbasi et al., 2021; Lo and Sim, 2021; Lubecke et al., 2021; Mukati et al., 2021; Mumtaz et al., 2021; Ni et al., 2021b; Peladarinos et al., 2021; Rehman et al., 2021; Tan et al., 2021; Uddin et al., 2021; Zhang et al., 2021; Dadon et al., 2022; Goergen et al., 2022)
Contact tracing	Indoor contact tracing	(Jeong et al., 2019; Dmitrienko et al., 2020; Kumar et al., 2022; Li et al., 2020b; Otoom et al., 2020; Schmidtke, 2020; Thangamani et al., 2020; Abueg et al., 2021; Lo and Sim, 2021; Mcheick et al., 2021; Wojtusiak et al., 2021)
	Outdoor contact tracing	(Elbir et al., 2020; Maghdid and Ghafoor, 2020; Ting et al., 2020; Gouissem et al., 2021; Gupta et al., 2021; Khatib et al., 2021; Shi et al., 2021; Tu et al., 2021; Yi et al., 2021; Malloy et al., 2022)
Machine learning framework and approaches	ML on IoT	(Karmore et al., 2022; Magesh et al., 2020; Maghded et al., 2020; Sathyamoorthy et al., 2020; Vedaei et al., 2020; Alsarhan et al., 2021; Barnawi et al., 2021; Ghayvat et al., 2021; Purnomo et al., 2021; Zuhair et al., 2021; Almalki et al., 2022; Dataset, 2022b,f; Fahad et al., 2022)
	Cloud and 5G	(Alanazi et al., 2020; Al-Qaness et al., 2020; Rustam et al., 2020; Ahmed et al., 2021; Al-Turjman, 2021; Andreas et al., 2021; Chegini et al., 2021; Muhammad et al., 2021; Singh and Kaur, 2021; Siriwardhana et al., 2021; Dataset, 2022a,c,d,e,g; Khelili et al., 2022; Mir et al., 2022)
	Multimodal datasets	(Wang, 2003; Canziani et al., 2016; Ke et al., 2017; Deshpande and Schuller, 2020; Islam et al., 2020; Li et al., 2020a; Vaishya et al., 2020; Chen et al., 2021; Dogan et al., 2021; Lv et al., 2021; Muguli et al., 2021; Orlandic et al., 2021; Schuller et al., 2021; Shorfuzzaman, 2021; Wang et al., 2021; Xia et al., 2021; Zhou et al., 2021; Dang et al., 2022; Deshpande et al., 2022; Han et al., 2022)
	ML models	(Vekaria et al., 2020; Wang et al., 2020; Adhikari et al., 2021; Awal et al., 2021; Elbasi et al., 2021; Jing et al., 2021; Kanmani et al., 2021)
Telemedicine		(Bahl et al., 2020; Gadzinski et al., 2020; Ganapathy et al., 2020; Iyengar et al., 2020; Kichloo et al., 2020; Lukas et al., 2020; Ohannessian et al., 2020; Omboni, 2020; Royce et al., 2020; Sufian et al., 2020; Alam and Rahmani, 2021; Elagan et al., 2021; Gupta et al., 2021; Hassan et al., 2021; Iqbal et al., 2021; Jiang et al., 2021; Kocsisné and Attila, 2021; Tahiliani et al., 2021; Awotunde et al., 2022)
Security		(Dataset, 2017; Al-Turjman and Deebak, 2020; Fernández-Caramés et al., 2020; McLachlan et al., 2020; Rahman et al., 2020; Sharma et al., 2020; Sufian et al., 2020; Alam and Rahmani, 2021; Fourati and Samiha, 2021; Hassan et al., 2021; Ng et al., 2021; Ni et al., 2021a; Singh et al., 2021; Sowmiya et al., 2021; Tahiliani et al., 2021; Aich et al., 2022; Jain et al., 2022; Kallel et al., 2022; Xu et al., 2022; Liu et al., 2023)

**Table 3:** Summary of selected papers in this survey as illustration examples.

**Row**	**References**	**Country**	**Journal/conference**	**Method or focus**	**Functionality**
1	Awal et al. (2021)	Bangladesh	IEEE Access	ML framework (Section 2.3.4)	Detection from inpatient data
2	Xu et al. (2022)	China	IEEE Transactions on Services Computing	ML and edge-cloud (Section 2.5)	Detection on X-ray images
3	Elbasi et al. (2021)	Kuwait	Electronics	ML algorithms (Section 2.1)	Public space monitoring
4	Omboni (2020)	Italy	Telemedicine and e-Health	Case study (Section 2.4)	Create interconnection and multidisciplinary research
5	Seshadri et al. (2020)	USA	Front. Digit. Health	Survey (Section 2.1.2)	Digital health platforms
6	Tahiliani et al. (2021)	India	IEEE Internet of Things Magazine	Blockchain (Section 2.4, Section 2.5)	Data security and user privacy
7	Lo and Sim (2021)	USA	Annals of Internal Medicine	Survey (Section 2.1)	Framework for assessing contact tracing
8	Mir et al. (2022)	India	Journal of Healthcare Engineering	IoT-enabled framework (Section 2.3.2)	Detection and prediction on IoT data
9	Tan et al. (2021)	USA	Neural Computing and Applications	5G and LSTM (Section 2.1.2)	Real-time cardiovascular monitoring system
10	Tu et al. (2021)	China	IEEE Sensors Journal	CNN for PDR positioning trajectory (Section 2.2.2)	Contact tracing
11	Jiang et al. (2021)	USA	IEEE Reviews in Biomedical Engineering	AI and sensor fusion (Section 2.4)	Long-term monitoring for chronic diseases
12	Orlandic et al. (2021)	Switzerland	Scientific Data	Fleiss' Kappa scores (Section 2.3.3)	Cough detection and classification
13	Jeong et al. (2019)	South Korea	IEEE access	Magnetometer-based diagnostic test (Section 2.2.1)	Automatic contact tracing
14	Vekaria et al. (2020)	USA	IEEE access	LSTM model (Section 2.1.1)	Health monitoring
15	Wang et al. (2020)	UK	IEEE access	Reinforcement learning (Section 2.3.4)	Risk-aware identification
16	Zhou et al. (2021)	China	Applied soft computing	CNN, Transfer learning (Section 2.3.3)	COVID detection and classification

### 2.1. Detection and monitoring

IoT devices have the ability to transmit data by connecting wirelessly to a network (Ding et al., 2020; Al-Emran and Ehrenfeld, 2021; Elbasi et al., 2021). Thus, wearable devices, such as head bands, chest bands, and wrist bands (Awotunde et al., 2021; Uddin et al., 2021; Goergen et al., 2022) have been used to collect real-time vital information from people to their smartphones for COVID-19 monitoring and detection. The focus of these technologies is on the health and wellbeing of occupants within a certain area.

The end goal is two-fold: monitoring and detection before and after the COVID-19 infection. Detection is for the purpose of timely treatment in the case when a patient becomes symptomatic. Monitoring is to further mitigate the spread of COVID-19 once the test is positive by emphasizing continuous monitoring of COVID-19 patients after testing positive (Arun et al., 2020; Mukati et al., 2021).

IoT devices are able to measure specific human physiological parameters such as heart rate (HR), respiration rate (RR) and blood pressure (BP), electrocardiogram (ECG), Electroencephalogram (EEG), electromyography (EMG), body temperature, Oxygen levels, blood glucose (BG), and the volume of air inspired and expired by lungs (Swayamsiddha and Mohanty, 2020; Chakkor et al., 2021b; Zhang et al., 2021), so that the early diagnosis and necessary treatment can be given One proposed system leverages IoT devices as part of its architecture to actively monitor users data and send this data to heath care centers for evaluation. Also, there are relatively new measurements that monitor the users' heart rate, number of daily steps and sleep time to scan pre-symptomatic cases of COVID-19 for the same purpose—early diagnosis and timely necessary treatment (Josephine et al., 2020; Lo and Sim, 2021; Ni et al., 2021b; Dadon et al., 2022). The IoT devices, as proposed, can work as both a precautionary measure for those that are not infected by COVID-19 as described above and a monitoring tool for those that are infected (Ashwin et al., 2021; Goergen et al., 2022). In the following, we present the monitoring approaches before infection and those after infection, respectively.

#### 2.1.1. Monitoring before infection

Wireless networks have been utilized for the purpose of detecting and monitoring COVID-19 infection. Many methods use data from a multitude of IoT devices including temperature sensors, camera data, and oxygen levels to detect and monitor a patient for COVID-19 (Choyon et al., 2020; Anjali et al., 2021; Awotunde et al., 2022). Researchers have also been seeking more advanced methods. One method uses a prototype that is developed from software-defined radios (SDRs) designed for 5G new radio wireless communications to monitor a consistent respiratory rate more accurately than devices used in common medical practice for the purpose of predicting COVID-19 infections (Lubecke et al., 2021). Another method integrates a temperature sensor into a smart phone based tracking system to get further information about a user's possibility of infection with COVID-19 (Karthi and Jayakumar, 2020). Some methods predict COVID-19 by utilizing CT-scan data and AI models and then tracking the patients to ensure that they are socially distancing and their vital signs, such as body temperature, are normal (Hossain et al., 2020).

One separate work creates a COVID-19 detection “network” which is achieved by distributing devices from local authorities to be imperatively worn by every citizen over the age of 14 years old and maintaining the network information in the cloud (Chakkor et al., 2021a). The device, an intelligent check strap, contains a body temperature sensor, cough sensor, heart rate sensor, blood oxygen sensor, and GPS sensor. The entire system is for the purpose of effectively locating wearers' GPS locations and detecting their health information to predict possible COVID-19 infection (Seshadri et al., 2020).

One avenue of research has been using IoT sensors, specifically air quality sensors, for detecting the prevalence of COVID-19 inside of buildings. To be more specific, the method uses sensors to sense various pollutants using artificial intelligence systems that are able to detect if there is COVID-19 pollutants inside of the air of the building and therefore an infected occupant (Mumtaz et al., 2021; Peladarinos et al., 2021).

#### 2.1.2. Monitoring after infection

Some research efforts have been focusing on a building's detection and monitoring platform in case of COVID-19 infection by utilizing a multitude of input movements and rhythms of patients, including heart rhythm as well as coughing, a spontaneous reflex, to detect COVID-19 and the severity of it. By measuring a patient's body condition, a device is able to predict and notify the increase or decrease in severity of the virus. Besides monitoring severity, there are monitoring tools combined with contact tracing to mitigate the spread of the virus (Rehman et al., 2021).

In case of predicting that the severity increases to the next stage, a device will alert the user for the need to advance their care (Dhadge and Tilekar, 2020). In addition to using preventive measures, data from wireless devices also has been used to aid the recovery of patients that have been infected with COVID-19. One example of this work utilizes artificial intelligence systems along with the data from wireless 5G devices to predict infected patients' cardiovascular health (Tan et al., 2021).

### 2.2. Contact tracing

COVID-19 is airborne and can spread in both indoor and outdoor environments. Unique methods have been utilized in indoor and outdoor settings for contract tracing to avoid COVID-19 spread. Due to their mobile nature, wireless devices have become a topic of research for the purpose of digital contact tracing to predict the spread of COVID-19 infection. One proposed system leverages IoT devices as part of its architecture to actively monitor users' data and send this data to health care centers for evaluation (Otoom et al., 2020). The IoT devices can detect symptomatic and asymptomatic patients for the indoor settings, outdoor settings both, which allows for easier contact tracing in case of a pandemic outbreak (Thangamani et al., 2020).

For a confirmed COVID-19 infection detection, systems can send out notifications to individuals with close contact to mitigate the COVID-19 spread. Abueg et al. (2021) proposed an agent-based model, a model simulating autonomous agents to learn the behavior of a system that is able to explore the disease dynamics within complex human interactions, social networks, and interventions. Using the model for the exposure notification within the 15% users of the overall population in Washington state's three counties, King, Pierce, and Snohomish, the infections and death was reduced by approximately 8 and 6% (Abueg et al., 2021). Outside of real-time detection and analysis, wireless devices were used during the peak of the COVID-19 pandemic in government labeled “hot-spots” to gather data for machine learning models that could be used to predict COVID-19 severity and recovery rates in patients (Kumar et al., 2022). Table 4 summarizes the papers on contact tracing reviewed in this survey. We primarily classify the contract tracing methods into two categories: indoor setting and outdoor setting, and present the research in each category in the following.

**Table 4:** Summary of the papers on contact tracing reviewed in this survey.

**Setting**	**References**	**Platform/application**	**Approaches**
	Wojtusiak et al. (2021)	Wifi	Five complex algorithms
	Dmitrienko et al. (2020)		Deterministic classifiers
	Mcheick et al. (2021)	Wifi-Direct	Statistical analysis
	Jeong et al. (2019)	Magnetometer	Pearson correlation coefficient
Indoor	Gouissem et al. (2021)	Wifi and Bluetooth	Mathematical models
	Abueg et al. (2021)	Individual-based model	Statistical analysis and ENS
	Thangamani et al. (2020)	Wifi and IoT	Machine learning
	kumar et al. (2003)	IoT	Machine learning
	Lo and Sim (2021)	Smartphone apps framework	Manual and digital
	Li et al. (2020b)	Household cohort study	Statistical analysis
	Yi et al. (2021)	Cellular	Deep neural networks
	Khatib et al. (2021)		Statistical analysis
	Shi et al. (2021)	Smartphone	Social network, mobility, Susceptible Exposed Infected Recovered (SEIR)
	Tu et al. (2021)	Wifi	Convolutional neural network
Outdoor	Malloy et al. (2022)		Contact graph
	Maghdid and Ghafoor (2020)	GPS	Clustering
	Elbir et al. (2020)	Vehicular network	Machine and federated learning
	Otoom et al. (2020)		Machine learning
	Gupta et al. (2021)	IoT	Machine learning and cloud computing
	Ting et al. (2020)		deep learning
	Schmidtke (2020)	Tracking application	Proximity, fingerprinting, and triangulation

#### 2.2.1. Indoor setting

Some methods utilize WiFi technologies (Mcheick et al., 2021; Wojtusiak et al., 2021) in indoor settings to track the density of population within a building or in certain areas of space. In this scenario, users do not have to carry devices with them. Rather, as Dmintrekno et al. show in their work (Dmitrienko et al., 2020), WiFi connected wireless devices within a building are utilized to detect the proximity of building occupants. This information allows for digital contact tracing in case of an initial infection of one of the occupants in the building (Dmitrienko et al., 2020). Specifically, each user's location, time and date are logged in the WiFi program once the users are connected. Once positive COVID-19 test is confirmed, it will report the test results to the WiFi program and use manual or automatic contact tracing to notify other users. In practice, users can pause tracing by turning off WiFi, so the names of users are not revealed (Lo and Sim, 2021). It should be noted that WiFi contact tracing is usually used in Universities and private companies, and households are usually seen as a whole entity as studies have found that the secondary transmission of COVID-19 in household is 16.3%, with 17.1% for adults compare to 4% for children, in non-quarantined situations (Li et al., 2020b).

Furthermore, there are other technologies to track populated areas to avoid COVID spread, such as a Smartphone Magnetometer. Once worn by individuals, the device is able to track where people are, see their distance relative to others, and alert people if certain areas are densely populated (Jeong et al., 2019).

#### 2.2.2. Outdoor setting

Research has been conducted on using cellular devices for digital contact tracing outside of buildings (Yi et al., 2021). One avenue of research has been utilizing smartphones and other mobile devices that are connected to cellular networks to track and monitor patients, with the aim of predicting the potential spread of COVID-19 in case of an infection breakout (Khatib et al., 2021; Shi et al., 2021). This includes using the smartphones onboard GPS signals for the purpose of contact tracing and predicting the spread of COVID-19 (Maghdid and Ghafoor, 2020; Tu et al., 2021). In addition to the GPS technology, the Bluetooth capabilities of the smartphone have been utilized along with WiFi signals to track users (Gouissem et al., 2021; Malloy et al., 2022). Vehicular networks, networks installed in public transport and other vehicles, have also been proposed for the purpose of better tracing and prediction of COVID-19 (Elbir et al., 2020).

Some researchers have worked on understanding how wireless devices in a connected ecosystem of devices can be utilized in the future to combat future pandemics such as the COVID-19 pandemic by proactively monitoring and predicting disease outbreaks. Gupta et al. (2021) proposed a smart community that consists of synergistic applications and technology systems for various smart infrastructures including E-Health, smart home, supply chain management, transportation, and city, which are “pandemic-proof” for future pandemics. The data collected from IoT devices can help public health agencies understand healthcare trends, model medical risk associations, and predict outcomes (Ting et al., 2020).

### 2.3. Machine learning framework and approaches

Machine learning models have been studied as tools for the purpose of COVID-19 infection prediction using data collected by various wireless devices ranging from specialized medical devices, to robot systems that are able to enforce social distance and scan for temperature (Sathyamoorthy et al., 2020), and common wireless smartphones. Research has also been done utilizing edge mobile IoT devices to run ML jobs directly on-device and transmit the data to the cloud or a 5G platform to do inference and receive results from them (Maghded et al., 2020; Ghayvat et al., 2021; Fahad et al., 2022). Furthermore, the data used by the research work is not only limited to the typical sensor data such as respiratory rate, but also includes multi-modal textual data and speech data. Here, data modality refers to the number of different types of data included in the dataset. In addition, the ML models used for detection and prediction in the research are quite diverse. Table 5 summarizes the different ML models, used variety of datasets, and metrics of the publications discussed in this paper.

**Table 5:** Summary of ML models, data modalities, and metrics of the papers reviewed in this survey.

**References**	**Journal/conference**	**Data**	**Data modality**	**ML**	**Metrics**
Magesh et al. (2020)	Iternation Journal of Pervasive Computing and Communications	Thermal, Acoustic	1-2	RNN, LSTM	N/A
Vedaei et al. (2020)	IEEE access	Health parameters	4	SVM, Decistion tree	Accuracy: 68.9–76.9%, F1-score: 69.7–77.3%
Purnomo et al. (2021)	Sensors	Breathing Movement	1	XGBoost, MFCC	Accuracy: 87.38%
Almalki et al. (2022)	Computing	UAV Thermal image	1	CNN, MANN	Accuracy: 82.63%, F-1 score: 0.98
Alsarhan et al. (2021)	International Journal of Interactive Mobile Technologies	Contact tracing data	1	RL	Packet loss probability: 0.1–0.4, Arrival rates: 80–120
Fahad et al. (2022)	Biomedical Engineering: Applications, Basis and Communications	CT images	2	AI-PSR model	N/A
Barnawi et al. (2021)	Future Generation Computer Systems	UAV Termal image	2	CNN, DCNN	Accuracy: 98–99.4%, Precision: 100%, 96–99%
Karmore et al. (2022)	IEEE Sensors Journal	Humanoid modules	6	Decision tree, TCN	Sensitivity: 95.39%, Specificity: 97.60%, Precision: 95.47%, Accuracy: 97.95%
Mir et al. (2022)	Journal of Healthcare Engineering	IoT Sensors	7	SVM, decision tree, NB, LR, NN	SVM Accuracy: 93.0%
Muhammad et al. (2021)	IEEE Network	Cough sound, Chest X-ray	2	FL	Accuracy: 95%, Precision: 97%-99%
Khelili et al. (2022)	Biomedical Signal Processing and Control	X-ray images	3	CNN	Classification: 97%, Precision: 100%
Singh and Kaur (2021)	World Journal of Engineering	Framework measurement	4	Fog computing	Classification: 81.2%, Kappa: 0.732, RMSE: 0.241
Alanazi et al. (2020)	Journal of healthcare engineering	COVID-19 data	3	Statistic analysis	N/A
Zhou et al. (2021)	Applied soft computing	CT images	2	CNN, Transfer learning, Ensemble learning	Accuracy: 97–99.05%
Shorfuzzaman (2021)	Computing	CT images	2	CNN	Accuracy: 96.58%, Precision & Specificity: 99.16%, AUC score: 96.6%
Orlandic et al. (2021)	Scientific Data	Audio dataset	7	XGB, CV	Precision: 95.4%, Sensitivity: 78.2%, Specificity: 95.3%, Balanced Accuracy: 86.7,% AUC: 96.4%
Xia et al. (2021)	NeurIPS	Audio dataset	6	SVM, CNN	AUC: 75% Sensitivity: 70%, Specificity: 70%
Dang et al. (2019)	Journal of Medical Internet Research	Audio dataset	3	GRU	AUC: 79%, Sensitivity: 75%, Specificity 71%
Ardabili et al. (2020)	Algorithms	COVID dataset	2	MLP, ANFIS	N/A
Vekaria et al. (2020)	IEEE Internet of Things Journal	IoT and economy data	5	LSTM	MAPE: 1.27%, RMSE: 6308
Wang et al. (2020)	IEEE Access	Social Internet of Things (SIoT) data	2	FL, GNN	N/A

#### 2.3.1. Machine learning on IoT devices

Widely-used smartphones, smart watches, and smart devices are promising in helping COVID-19 detection and prediction using ML techniques. One method utilizes a patient's temperature data from an infrared thermometer, a thermographic camera, and an acoustic device as inputs to a Mode and Mean Missing Data Imputation (MMM-DI) method to predict COVID-19 infections through recurrent neural network (RNN) with long-term short memory enacted (Magesh et al., 2020). Another system utilizes smartphone apps and the patients' IoT devices to track and send vital signs to an ML model in the cloud that predicts the risk for COVID-19 infection in real time in an area and then notifies the users (Vedaei et al., 2020). This approach is also utilized on a patients' breathing patterns for predicting COVID-19 detection through Xtreme Gradient Boosting (XGBoost), a gradient boosting machine (GBM) algorithm, and classification ML models (Purnomo et al., 2021). Some ML-based approaches use smart devices [e.g., Whoop (Dataset, 2022f), Aura (Dataset, 2022b)] for COVID-19 infection detection using their respiratory rate. Whoop is a wearable that has been validated by a third party clinical trial to accurately measure respiratory rates to predict COVID infection (Dataset, 2022f). Furthermore, there is a combination of AI and physiological sensor readings (AI-PSR) that helps the doctors predict and diagnose COVID based respiratory failures (Fahad et al., 2022).

One area of research focuses on integrating drones and Unmanned aerial vehicles (UAVs) into the wireless architecture along with AI systems to predict and combat COVID-19 (Alsarhan et al., 2021). The approach uses thermal imaging cameras on the UAVs to evaluate body temperatures for helping minimize the risk of spreading the infection through close contact (Barnawi et al., 2021; Almalki et al., 2022). The drones are also equipped with face mask recognition system to detect whether a person has a mask on the face or not (Barnawi et al., 2021; Zuhair et al., 2021). Another area of research focuses studying integrating AI systems with humanoid robots to detect COVID-19 in potential patients. The humanoid, through real time monitoring by AI system, is able to provide a patient with a complete COVID diagnosis, which reduces the risk of spreading virus and medical system workload (Karmore et al., 2022).

#### 2.3.2. Cloud and 5G platforms

Mir et al. (2022) proposed a framework consisting of data collection center, data analytic center, diagnostic system, and cloud system (Mir et al., 2022). Through data collection and data analysis, the information would be stored into the cloud and be reused by healthcare professionals for further analysis. Also, the data will be used to update machine learning models for deriving more accurate results.

Research has also been conducted toward integrating AI systems with 5G networks and the cloud. The fifth generation cellular technology assists medical services with 5G-cloud Robots that lessen the workload on mobile devices (Ahmed et al., 2021; Muhammad et al., 2021; Siriwardhana et al., 2021). The robot can be used for disinfection of surfaces, temperature testing, food delivery, and medicine delivery to infected peoples to mitigate COVID-19 virus spread.

Some methods deploy AI and ML systems onto the cloud for the purpose of COVID-19 prediction (Al-Turjman, 2021; Mir et al., 2022). Server or cloud based AI screening services for COVID-19 such as those in Bouy Health (Dataset, 2022g) and others (Dataset, 2022c,d,e) have been developed. In these screening systems, the ML models running in the cloud receive data from users and send screening results back to the users. Some other works design fog-cloud based systems for COVID-19 prediction using IoT devices (Singh and Kaur, 2021; Khelili et al., 2022), where the fog computing cluster enables the processing of IoT tasks independent to the Cloud layer (Chegini et al., 2021).

AI and ML models and systems have also been integrated with Geo-locating systems to predict and model the potential spread of COVID-19 (Andreas et al., 2021). In a similar vein, ML models also have been used during the peak of the pandemic to forecast a long-term trajectory of the spread of the virus (Alanazi et al., 2020; Al-Qaness et al., 2020; Rustam et al., 2020).

#### 2.3.3. Multimodal datasets

As promising tools, ML and AI techniques are considered as a key element in epidemic and transmission prediction, diagnosis and detection, and development for new treatment options. These models utilize datasets that are based on patients' basic information, patients' health and medical data, and the COVID-19 test results and. Three main types of datasets in COVID-19 have been gathered and used: textual data, medical data and speech data. Textual data includes dashboard, mobility data, case reports, social media posts, and articles. Medical data is from the diagnosis and screening of COVID-19 patients such as medical X-rays, CT scans, ultrasound or MRI. Speech data includes cough sound, breathing rate and stress detection techniques (Dogan et al., 2021). It should be noted that missing value detection is necessary for pre-processing (Wang et al., 2021) for all data that is used.

Chest X-ray images, CT images, mobile sensor data, COVID-19 symptoms (Islam et al., 2020), and previous medical data of the patients are used as input for an ML method to predict COVID-19 (Vaishya et al., 2020). AI and ML methods have a significant contribution in the areas of vaccine discovery, in which the development focuses on the prediction of potential epitopes by using a variety of methods including artificial neural network, gradient boosting decision tree and deep neural network (Wang, 2003; Canziani et al., 2016; Ke et al., 2017). AI has been used in combating COVID-19 from the aspects of COVID-19 detection and diagnosis, virology and pathogenesis, COVID-19 drug and vaccine development, and epidemic and transmission prediction in COVID-19 (Chen et al., 2021; Lv et al., 2021).

One example of research that has been conducted using medical data to predict COVID-19 infection utilizes patient's CT scan images (Zhou et al., 2021). CT images can reflect clinical COVID-19 classification because of the high consistency and high diagnostic ability. Shorfuzzaman (2021) proposed an IoT-enabled end-to-end integrated stacked deep learning method to precisely detect COVID-19 infections using CT images. It uses three different fine-tuned CNN models and achieves accuracy of 96.58% for the categorization of COVID-19 and non-COVID CT images with a high value of specificity. However, using chest CT images alone can lead to misdiagnosis of severe COVID-19 patients, which would bring potential infection risk; therefore, CT images were not recommended for an independent screen tool (Li et al., 2020a).

Audio signal analysis is another promising technique to determine overall health status of individuals. Xia et al. (2021) and Muguli et al. (2021) proposed a study using large-scale respiratory audio dataset with wide variety of demographics and health conditions to explore the potential of affordable health status prediction through audio-based machine learning method. Orlandic et al. (2021) developed COUGHVID, a cough audio dataset that includes over 25,000 crowdsourced cough recordings representing participants with different ages, gendersm and COVID-19 status. The dataset is partially labeled and used for audio classification tasks. Although the technology makes it feasible to monitor audio signals like cough, sneeze, breathing, and speech, there is a need of validation by performing gold standard test like chest CT or X-ray analysis by experts to have close-to-zero false negative rates for using such a tool for combating COVID-19 (Deshpande et al., 2022). Jing et al. selected 5,240 samples from 2,478 English-speaking participants and split them into participant-independent sets for model development and validation and the unbiased model yielded an AUC-ROC of 0.71 (Han et al., 2022).

The critical issue of using audio analysis is distinguishing COVID-19 cough from other illnesses, and tracking disease progression characteristics and patterns of recovery (Muguli et al., 2021; Schuller et al., 2021). Deshpande et al. emphasized that identifying the markers of COVID-19 in speech and other human generated audio signals requires other methods using along. For example, by using chat-bots from Microsoft, Apple, or other health system, it can differentiate over 20,000 diseases and is able to identify COVID-19 from other diseases with an accuracy of 96.32% (Deshpande and Schuller, 2020). Dang et al. explored the potential audio samples over time for COVID-19 progression prediction and recover pattern with test results, yielding an AUC-ROC of 0.79, and it suggests that monitoring COVID-19 evolution *via* longitudinal audio data is beneficial in the tracking of individuals' COVID-19 progression and recovery (Dang et al., 2022).

#### 2.3.4. Machine learning models

To reduce hospital admission pressure related to COVID-19, AI-assisted edge computing systems use edge-centric e-healthcare models for monitoring patient symptom to predict the risk levels according to the monitored symptoms (Adhikari et al., 2021). Also, a variety of COVID-19 prediction models have been proposed, ranging from decision trees, Naive Bayes classifier, adaptive network-based fuzzy inference system, Multi-Layer perceptrons, and Support Vector Machines (Ardabili et al., 2020). These models have been designed to run on edge devices (Adhikari et al., 2021) as well as on the cloud (Awal et al., 2021; Elbasi et al., 2021; Jing et al., 2021), with some models on the cloud utilizing stored data such as temperature data, audio data, and heart rate data (Kanmani et al., 2021) to make COVID-19 diagnoses. Deep learning methods also have been utilized to predict COVID-19 infection using the data gathered from wireless devices. One example of this work is a learning pipeline that utilizes data from IoT devices to predict COVID-19 infections using a Long Short-Term Memory (LSTM) (Vekaria et al., 2020). Furthermore, another approach uses reinforcement learning COVID infection data to build neural networks capable of predicting COVID-19 (Wang et al., 2020).

### 2.4. Telemedicine

Telemedicine is the treatment of patients remotely using wireless devices, such as telecommunication devices. It has been a rising trend because of its convenience and the popularity of the use of IoT devices (Bahl et al., 2020). More recently, due to the need of reducing face-to-face consultations in the COVID-19 pandemic, telemedicine systems have been implemented globally (Ganapathy et al., 2020) as with telemedicine, a patient does not need to go to a heath-care center (e.g., hospital) to get a diagnosis, reducing the risk of spreading the virus by face-to-face interaction (Lukas et al., 2020).

As a result, there has been a body of research conducted on the application of telemedicine for remote prediction and consultation of COVID-19 using IoT medical devices (Ohannessian et al., 2020; Kocsisné and Attila, 2021). Many examples of the technologies to monitor patients and detect COVID-19 early are based on the uses of traditional smart medical sensors that measure the patients' pulse, thermal information, and blood oxygen level (Elagan et al., 2021; Iqbal et al., 2021). Jiang et al. analyzed existing wearable devices and proposed a wearable tele-health solution for monitoring a set of physiological parameters that are critical for COVID-19 patients. This new device shows promising results with performance comparable to or better than similar commercial devices, which makes the proposed system an ideal wearable solution for long-term monitoring of COVID-19 patients and other chronic diseases. These devices can still be utilized after patients are detected with COVID-19 positive, so their health condition can still be monitored virtually (Jiang et al., 2021). With the possibility of using telemedicine devices to predict, diagnose, control, and track patient conditions, the pressure and tension on healthcare professionals can be reduced. At the same time, it potentially can eliminate medical faults, reduce workload (Kichloo et al., 2020), increase medical staff productivity, reduce long-term healthcare costs, enhance patient satisfaction during COVID-19 pandemic. The telemedicine treatments can be extended in other areas such as cancer, neurology, and urology and so on (Gadzinski et al., 2020; Ganapathy et al., 2020; Royce et al., 2020; Awotunde et al., 2022).

It should be noted, however, that although telemedicine has been an effective solution of delivering healthcare while keeping everybody safe, it is not without its faults that need to be ironed out going into the future. Telemedicine has three main challenges. First, telemedicine services need to be connected between national health systems and low level primary care clinics or community pharmacies so the patients' information is always updated (Omboni, 2020). Second, appropriate training will be needed for medical staffs doing remote consultation in clinical practice, and patients need to be informed on how to use telemedicine technology to provide doctors with the information they need (Iyengar et al., 2020). Third, patients' data privacy is imperative to be secured. It will need engineering effort to integrate technology such as federated learning, and blockchain (a back-linked database with cryptographic protocols) to achieve the goal (Sufian et al., 2020; Alam and Rahmani, 2021; Hassan et al., 2021; Tahiliani et al., 2021). With telemedicine and related IoT devices fully equipped, health systems can be more prepared for COVID-19 and other future pandemics (Gupta et al., 2021).

### 2.5. Security

The data that is used for diagnosis and tracking of patients for combating COVID-19 is often extremely sensitive and any leakage of the data would severely compromise the privacy of the patients. As a result, one area of research in the field of using wireless devices for COVID-19 prediction has been data privacy and security. Research has been conducted on evaluating the current security risks that exist with using patient data for the purpose of contact tracing and it found that there are a multitude of attacks that are possible in contact tracing (Sowmiya et al., 2021). These attacks range from wireless device tracking, denial of service attacks, enumeration attacks that count the number of people infected with COVID-19, and blue-snarfing attacks that hijack the Bluetooth connection and obtain personal data including pictures and videos on a users phone (Sowmiya et al., 2021). There also has been work that demonstrates that learning models are prone to security issues as well through adversarial examples (AEs), which are intentionally designed inputs to cause the ML model to make mistakes (McLachlan et al., 2020; Rahman et al., 2020).

One solution for some of the security issues is integrating IoT devices with the blockchain technique (Fernández-Caramés et al., 2020; Singh et al., 2021; Jain et al., 2022). A blockchain is a shared, in-mutable ledger that facilitates the process of recording transactions and tracking assets in a business network (IBM, 2022). Block chain integrated federated learning has been proposed to effectively build COVID-19 predicting models without compromising privacy (Aich et al., 2022). Blockchain technology is also able to improve clinical trial data management by reducing delays in regulatory approvals, and streamline the communication between diverse stakeholders with immutability, decentralization, and transparency. The integration of block chain technology has also been researched for the purpose of contact tracing during COVID-19, and it offers enhanced data security and functionality without compromising user privacy at reasonable blockchain transaction fee (Tahiliani et al., 2021) which ensuring that the information received is more reliable and trustworthy at low cost.

Abhishek et al. proposed to use blockchain-based platform to combat the COVID pandemic that provides improved solutions, outbreak tracking, user privacy protection, performance of the medical supply chain, donation tracking, and safe day-to-day operations (Ng et al., 2021). Dounia et al. presented a blockchain-based system using Ethereum smart contracts and oracles to track COVID-19 related data from trusted source with economical feasibility to ensure data integrity, security and traceability among stakeholders (Sharma et al., 2020). Similarly, Liu et al. (2023) implemented a COVID-19 contact tracing scenario framework based on blockchain that protects the privacy of close contacts with some cryptographic tools. Also, by using aggregated signatures, the verification is more efficient. The authors suggested using public and private locations for better tracing solutions with higher-level security.

Another avenue of research exploring solutions to many of these security issues in using wireless devices for predicting and monitoring COVID-19 is minimizing the number of communications between a device and medical center through a mobile sink (a mobile node that connects the sensor network to an existing communication infrastructure with minimal energy and delay) (Al-Turjman and Deebak, 2020). Another avenue of work is illustrated by Ni et al. who proposed LPP-SVM-PI, a labeling privacy protection support vector machine that enhances the security of the labeling scheme while maintaining the classification performance (Ni et al., 2021a).

Federated learning has also been researched to solve some of the security issues (Xu et al., 2022). In federated learning, the data is no longer shared between each device and a central server. Rather, it is on the device that collects the data. The devices send their trained model parameters to the central server, and the trained model is then shared among various devices Dataset, 2017). This architecture of learning has been a focus for COVID-19 infection prediction due to the fact that many medical devices such as wrist bands are edge devices which can transmit data to smart phone and process the information (Hassan et al., 2021). Federated learning has been utilized for both traditional learning as well as transfer learning (Sufian et al., 2020; Alam and Rahmani, 2021). To provide further security protection and scalability, research has been conducted in integrating federated learning systems into the cloud. The traditional fog based architecture enhances the system's responsiveness to events by processing the data in the site, while the cloud layer offers good performances for deep learning and machine learning techniques. Federated learning takes advantage of both architectures and also enhances the security and performance at the same time (Fourati and Samiha, 2021; Kallel et al., 2022).

## 3. Future work and technical challenges

Although there has been a great amount of research work conducted on the topic of using wireless devices for the purpose of predicting COVID-19, there are still many challenges that lay ahead.

One challenge is data privacy. Due to the fact that a majority of the data collected in this case is often sensitive medical information, it is imperative that any usage of this data is done in a secure way without compromising the privacy of the patient. Future directions of work to address this challenge include the use and integration of technologies such as differential privacy, which is a privacy technique that uses random noise to ensure that the public record doesn't change if one record in the dataset changes (Müftüoğlu et al., 2020), Federated Learning, and the blockchain. Even with these robust technologies, the challenge remains within effectively using these techniques to provide stronger security and privacy protection as illustrated by Hauer and Santos-Lozada (2021); who found that using differential privacy will sometimes introduce substantial distortion in COVID-19 mortality rates, causing mortality rates to exceed 100 percent and hindering our ability to understand the pandemic. Therefore, there is a need of balancing data privacy and utility.

One avenue of future work that lays ahead in this line of COVID-19 detection and prediction work is the integration of the proposed detection technologies into the current healthcare and city infrastructure. Although the work and research being conducted in this field is revolutionary and beneficial, much of this work has yet to be deployed on a large scale. The gap between theory and practice has yet to be closed in this field.

Another avenue of future work is to use AI power to minimize interactions between healthcare workers and patients in all phases while keeping a high accuracy of diagnosis. Optimizing the benefits of AI in health care will require a balanced approach that enforces accountability and transparency while promoting innovation. There are challenges of improving the accuracy of the AI diagnosis and reducing the false negative diagnosis rate. For COVID-19, researchers may combine chest imaging with clinical symptoms, exposure history, and laboratory tests in the diagnosis of COVID-19.

Finally, Smart cities and buildings need more attention since more effective and broader smart city initiatives can improve how critical data is retrieved, processed, stored and disseminated, potentially improving outbreak detection and mitigation while reducing the execution time to act. This is a promising solution against the devastating effects of the COVID-19 pandemic. However, there is no golden rule for building smart cities yet. Each city has to consider its unique situations before implementing certain technologies and systems (Costa and Peixoto, 2020).

## 4. Conclusion

In this paper, we present a survey of the current research work on the use of mobile IoT devices, AI and Telemedicine for the purpose of COVID-19 detection and prediction. In this paper, we first introduce monitoring and detection methods and their purposes, and then the contact tracing methods that can reduce the spread of the virus. We also present machine learning based approaches to aid combating the COVID-19 pandemic. Next, we present the use of telemedicine as a new approach for pandemics diagnosis. Lastly, we present security methods for privacy protection of users in combating COVID-19. COVID-19 is here to stay, and we must use all of the tools we can to effectively combat and mitigate its impact on our daily lives. Although there has been a great amount of research work done, there are still many challenges that lay ahead. Thus, we also illustrate the future work and challenges in using mobile IoT devices for combating COVID-19.

## Author contributions

All authors contributed to producing the paper. All authors contributed to the article and approved the submitted version.

## Funding

This research was supported in part by U.S. NSF Grants NSF-1827674, NSF-1822965, FHWA Grant 693JJ31950016, Microsoft Research Faculty Fellowship 8300751, and the Commonwealth Cyber Initiative (CCI) (2021UVA-02.005, HV-4Q21-001, and H-4Q21-011), an investment in the advancement of cyber research, innovation and workforce development. For more information about CCI, visit cyberinitiative.org.

## Conflict of interest

The authors declare that the research was conducted in the absence of any commercial or financial relationships that could be construed as a potential conflict of interest.

## Publisher's note

All claims expressed in this article are solely those of the authors and do not necessarily represent those of their affiliated organizations, or those of the publisher, the editors and the reviewers. Any product that may be evaluated in this article, or claim that may be made by its manufacturer, is not guaranteed or endorsed by the publisher.
